# Cytokines That Serve as Embryokines in Cattle

**DOI:** 10.3390/ani11082313

**Published:** 2021-08-05

**Authors:** Alan D. Ealy, Savannah L. Speckhart, Lydia K. Wooldridge

**Affiliations:** 1 Department of Animal and Poultry Sciences, Virginia Tech, Blacksburg, VA 24061, USA; slspeckhart@vt.edu; 2 The Jackson Laboratory, Bar Harbor, ME 04609, USA; Lydia.wooldridge@jax.org

**Keywords:** bovine embryo, blastocyst, pregnancy, embryokine, cytokine

## Abstract

**Simple Summary:**

This review will explore how some cytokines also influence early embryonic development. We term these types of molecules as embryokines. Understanding how cytokines serve as embryokines could offer new opportunities to improve embryo development and the overall health of the embryo so that pregnancies will be retained after embryo transfer and so that viable offspring are produced. At least two cytokines may offer these benefits to bovine embryos produced in vitro. Additional cytokines also are identified in this review that may contain beneficial activities on bovine embryos.

**Abstract:**

The term “embryokine” has been used to denote molecules produced by the endometrium, oviduct, or by embryo itself that will influence embryo development. Several cytokines have been identified as embryokines in cattle and other mammals. This review will describe how these cytokines function as embryokines, with special emphasis being placed on their actions on in vitro produced (IVP) bovine embryos. Embryokines are being explored for their ability to overcome the poor development rates of IVP embryos and to limit post-transfer pregnancy retention efficiencies that exist in IVP embryos. This review will focus on describing two of the best-characterized cytokines, colony-stimulating factor 2 and interleukin 6, for their ability to modify bovine embryo quality and confirmation, promote normal fetal development, and generate healthy calves. Additional cytokines will also be discussed for their potential to serve as embryokines.

## 1. Introduction

The dairy and beef industries are rapidly adopting the use of in vitro embryo production to produce calves of a desired genetic makeup. This is being achieved by coupling in vitro production (IVP) with ovum pickup (OPU) and embryo transfer (ET). These combined technologies maximize the number of calves that can be produced from genetically desirable dams and sires. Over 1 million transferrable IVP bovine embryos were produced globally in 2019 [1]. This represents a 2.5-fold increase in IVP bovine embryo production within the past 10 years.

**Figure 1:**
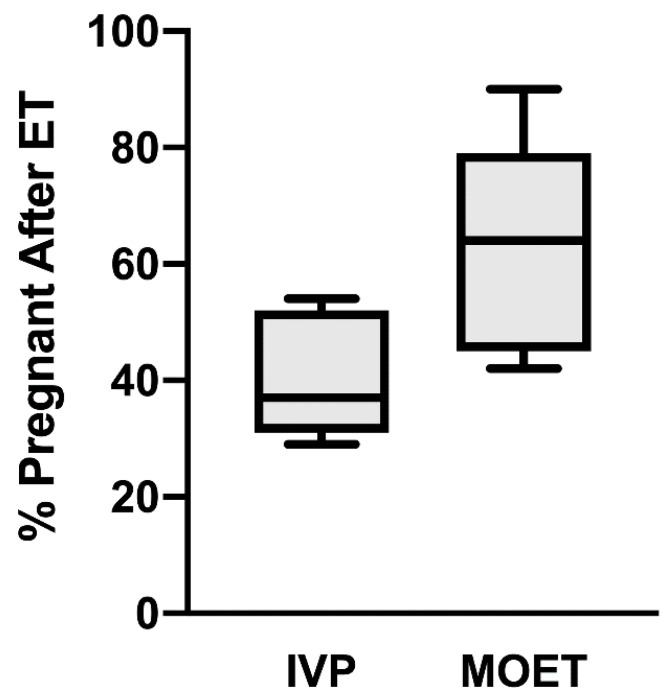
Differences in pregnancy retention after embryo transfer (ET) of in vitro-produced (IVP) embryos or embryo transfer of in vivo-produced embryos (termed MOET). Both IVP and MOET were evaluated within the same study in seven independent studies. Day of pregnancy detection ranged from day 30 to term. Six studies used *Bos taurus* cattle, and one study used *Bos indicus* cattle. In vivo-produced embryos were generated by superovulation, AI and uterine flushing at d 7 post-breeding. Each box contains the minimum and maximum values for each group, and the mean is represented by the line within the box. The bars represent SEMs. Adapted from [5].

The generation of calves from IVP bovine embryos has been possible since 1981 [2], but in vitro embryo production systems suffer from two primary constraints. First, the efficiency of embryo production remains low. Embryo production rates can vary drastically depending on various factors, including media formulation, culture conditions (e.g., atmospheric oxygen tension), season, and technical expertise. However, it is generally accepted that only from 20 to 45% of oocytes entering in vitro embryo production systems will generate transferrable, blastocyst-stage embryos [3,4]. Second, a substantial subset of transferable IVP embryos will not survive after ET. A recent review of the literature found that only 27% of ETs using IVP embryos resulted in calves in dairy and beef cattle [5]. As illustrated in Figure 1, the percentage of pregnancies that result after ET is approximately 20% lower in cattle receiving an IVP embryo than in cattle receiving an in vivo-generated embryo from a superovulated donor cow. Most of these IVP pregnancy failures occur during the first month of gestation [5]. This suggests that a subset of IVP embryos is not properly programmed to elongate, signal their presence to the maternal system, or achieve adequate placentation.

Developmental problems also exist in IVP-generated pregnancies that survive past the first month of gestation. Alterations in epigenomic profiles are seen in various organs of some IVP fetuses [6,7,8,9], increased incidences of dystocia occur in IVP pregnancies [10], and IVP-derived neonates have increased morbidity and mortality rates and an increased incidence of congenital abnormalities [10,11]. However, in terms of productivity, IVP-derived calves that survive the first few months of life appear to be no different from calves generated through AI. No differences are detected in age at first service, pregnancy rate at first service, and milk yield and composition between IVP and AI-derived heifers [6,10].

One way we may be able to improve in vitro embryo production efficiency and post-transfer IVP embryo competency is by supplementing media formulations with biologically active molecules produced by the reproductive tract in early pregnancy. Hansen and colleagues introduced the term “embryokine” to describe molecules produced by the oviduct, uterus, or embryo that regulate embryo development [12]. Various growth factors and signaling factors have been identified as embryokines. For example, insulin-like growth factor 1 (IGF1) supplementation improves IVP blastocyst development rates, blastocyst cell number, and post-transfer pregnancy rates during summer months in dairy cows [13,14,15]. Supplementing multiple embryokines can also produce additive responses on in vitro embryo production. For example, providing epidermal growth factor (EGF), fibroblast growth factor 2 (FGF2), and IGF1 during in vitro bovine embryo culture has a greater impact on blastocyst formation and blastomere numbers than when using a single embryokine [15].

This review will focus solely on describing the actions of embryokines that were first recognized as cytokines. Cytokines (“cyto” for cell, “kinos” for movement) are a broad category of intracrine, autocrine, paracrine, and endocrine signaling agents that regulates the innate and adaptive immune responses [16]. These molecules include chemokines, interferons, interleukins, tumor necrosis factors, and lymphokines. Various non-immune actions of cytokines are known, and this review will highlight the embryokine activities of these cytokines.

## 2. Cytokines of Interest and Their Signaling Pathways

Two families of cytokines have been explored as embryokines in cattle and other mammals. The first is the β-common cytokine family. Three members exist in this family: colony-stimulating factor 2 (CSF2; granulocyte-macrophage colony-stimulating factor [GM-CSF]), interleukin 3 (IL3), and IL5. This family is so-named because each of these cytokines utilizes a shared β-subunit receptor coupled with a cytokine-specific α-subunit receptor [17]. These factors and their receptors mediate Signal Transducer and Activator of Transcription 5 (STAT5) activation, but they also stimulate other intracellular signaling molecules, including SRC family kinases, phosphoinositide 3-kinase (PI3K), and mitogen-activated protein kinases (MAPK) [18]. All three members of this family have been examined for their ability to mediate embryo, conceptus, and placental activity in ruminants, but as discussed in greater detail below, CSF2 has received the greatest attention for its actions as an embryokine in the bovine embryo.

The second family of cytokines are members of the interleukin 6 (IL6) family. These cytokines are structurally and functionally distinct from the β-common cytokines. Members of the IL6 family include IL6, IL11, IL27, IL31, leukemia inhibitory factor (LIF) oncostatin-M (OSM), and ciliary neurotropic factor (CNTF) [19,20]. Each member of this family utilizes receptors that contain a cytokine-specific, α-subunit receptor coupled to a common β-subunit receptor that was originally termed glycoprotein 130 (gp130) but is now referred to as IL6 signal transducer (IL6ST) [21]. IL6ST is structurally and functionally different from the β-common receptor used by CSF2, IL3, and IL5. Signaling molecules used by IL6 family cytokines include STAT3, MAPK, PI3K, and nuclear factor-kappa B (NFkB) [20]. The molecules that have received the greatest attention as embryokines within the IL6 family are IL6 and LIF.

The following sections will explore the embryokine-related actions for CSF2, IL6, and LIF, as well as additional cytokines that may have the capacity to serve as embryokines.

## 3. Colony Stimulating Factor 2

There is evidence that CSF2 supplementation will improve IVP blastocyst development in the mouse [22], pig [23,24], and human [25], but it is less clear whether CSF2 contains this activity in bovine embryos. Some of the initial work on CSF2 supplementation found that IVP bovine blastocyst formation could be improved with CSF treatment [26,27,28], but more recent work failed to consistently detect these effects [29,30,31,32]. One general observation is that CSF2 is more likely to improve blastocyst formation when development rates are low, suggesting that CSF2 may only function when embryos are under some type of developmental or environmental stress that limits developmental potential. Another explanation for this disparity in outcomes could stem from the concentrations of CSF2 tested and from the source of the commercial CSF2 preparations used for these various studies. The initial studies utilized the same recombinant bovine CSF2 preparation, whereas the more recent studies used recombinant bovine CSF2 proteins from other companies. Unfortunately, the first recombinant CSF2 preparation is no longer available, so the reason why this particular recombinant protein preparation produced distinct biological responses from others remains unclear. However, although these newer recombinant proteins routinely failed to improve IVP blastocyst development, several other benefits of supplementing these CSF2 preparations have been noted, including improvements in IVP embryo cryo-survival [33], increased resistance to heat shock [34], increased inner cell mass (ICM) cell numbers and ICM:trophectoderm (TE) ratios [26,35], reduced apoptosis of ICM cells [30], and improved ICM cell pluripotency in extended cultures [30]. Additionally, these new CSF2 protein preparations alter the expression of >900 genes in the ICM and >800 genes in the TE at the blastocyst stage [36].

There is also compelling evidence that CSF2 improves IVP embryo competency after ET in cattle. Several large-scale ET studies detected improvements in overall pregnancy retention rates and/or reductions in late embryonic and fetal losses in pregnancies derived from CSF2-supplemented IVP bovine embryos [26,35]. Additionally, CSF2 treatment does not affect calf birth weight, but calves derived from CSF2-treated embryos have greater postnatal growth rates than calves from non-treated IVP embryos [37]. The beneficial effects of CSF2 supplementation on pregnancy outcomes are also evident in the mouse [22], and benefits of CSF2 supplementation prior to transfer have been noted in human recipients in one study [38] but not another [39].

There is one report in cattle that failed to detect a positive effect of CSF2 supplementation on post-transfer pregnancy outcomes [29]. This study was completed in a commercial setting, and the lack of CSF2 effect may have occurred because fetal bovine serum (FBS) was included in the commercial culture medium formulation. The addition of FBS is common in commercial IVP laboratories because it improves IVP embryo production efficiency, and the outcome of this work may indicate that a factor within FBS can mimic the effects of CSF2. Unfortunately, if this was the case, one should not rely on there being similar effects in every batch of FBS. The composition of FBS is highly variable from batch to batch, and the concentration of this factor or set of factors could differ dramatically in different FBS preparations. Therefore, further investigation is needed to better explore the potential benefits of CSF2 supplementation to commercial IVP embryos.

The ways that CSF2 improves post-transfer IVP embryo competency have not been fully described, but several key developmental processes are influenced by CSF2, and improvements in any one of these processes could, at least conceptionally, lead to improved pregnancy retention rates. One way that CSF2 may act is by improving the development and elongation of the peri-implantation conceptus (i.e., the embryo proper and its extraembryonic tissues). A greater percentage of elongating conceptuses were developing normally at day 15 post-ovulation from CSF2-supplemented IVP embryos than untreated embryos [40]. Furthermore, CSF-treated conceptuses were larger than the control conceptuses, and corresponding interferon-tau (*IFNT*; the maternal recognition of pregnancy factor in ruminants) concentrations were greater in flush solution from CSF2-treated conceptuses [40]. There are conflicting reports whether CSF2 supplementation increases *IFNT* expression in bovine embryos and conceptuses [31,40,41,42], so it remains unclear if the increase in uterine *IFNT* content occurs solely because CSF2-treated conceptuses contain a greater number of TE cells, the tissue that produces *IFNT*, or if CSF2 may also directly influence *IFNT* expression. Nonetheless, these findings indicate that CSF2 improves conceptus survival and *IFNT* production around the time of maternal recognition of pregnancy.

A very interesting facet of CSF2 action is that bovine embryos also respond differently to CSF2 based on their sex. CSF2 treatment increases blastocyst development for female embryos but not male embryos, even though CSF2 is equally effective at increasing ICM cell numbers in both sexes [43]. Differential effects of CSF2 are also noted on day 15 conceptuses, where CSF2 decreased conceptus length and uterine *IFNT* content in female IVP embryos but increased conceptus length and *IFNT* content in CSF2-supplemented male IVP embryos [42]. Reasons for the opposing effects remains unclear, but male and female conceptuses have distinct transcript profiles and DNA methylation patterns [42,43], and this likely provides CSF2 with the opportunity to differentially affect embryos and conceptuses based on sex. The implications of these sex-dependent changes on embryo competency have not been explored further.

One set of studies explored how CSF2 supplementation before ET affects fetal development at day 86 of gestation [44,45]. CSF2 supplementation did not affect the prevalence of large for gestational age fetuses, but it did influence the expression of selected genes within the liver, muscle, and placenta so that CSF2-treated IVP pregnancies more closely resembled AI pregnancies than non-treated IVP pregnancies [44]. Similarly, CSF2 exposure before ET modified a subset of differentially methylated regions in fetal liver and muscle, so the methylation patterns of IVP-derived fetuses more closely resembled those of AI-derived fetuses [45].

To summarize thus far, CSF2 may not resemble a conventional embryokine as it does not greatly impact IVP blastocyst formation. However, there is compelling evidence that CSF2 supplementation improves post-transfer pregnancy retention and calf health. What remains largely unclear is how CSF2 reprograms the IVP bovine embryo, so it is better able to survive after transfer. In fact, it remains unclear mechanistically how CSF2 exerts its actions within the bovine embryo. Bovine embryos express the CSF2-specific α-subunit receptor (termed CSFR2A) but do not express the β-common subunit (termed CSF2RB) [26,46]. The β-common subunit receptor is also absent in pig, mice, and human embryos [23,47,48]. The lack of this receptor subunit indicates that CSF2 must utilize an unconventional signaling system to exert its biological activities in embryos. This alternate signal system has not been discovered, but recent findings indicate that CSF2RA is required for CSF2 activity in bovine embryos [47].

## 4. Interleukin 6

The bovine oviduct and endometrium as well as the embryo produce IL6 in pregnancy [48,49,50], and the bovine embryo contains both the IL6-specific α-subunit receptor, IL6R, and the shared β-receptor, IL6ST [49,50]. Only a subset of the studies that explored the effects of IL6 supplementation on IVP blastocyst development detected any effects of IL6 on blastocyst development [49,50,51,52]. The only circumstance when IL6 supplementation improved embryogenesis occurred when IL6 was supplemented to individual-cultured embryos [49]. Blastocyst rates were still low in IL6-supplemented embryos (9.2% versus 21.0% with IL6 versus group-cultured controls), but none of the non-treated individually cultured embryos went past the 8–16 cell stage. This may indicate that IL6 promotes embryo survival; however, a lack of IL6 effect in group culture suggests either that other embryo-derived factors can substitute for IL6 in this role.

We have classified IL6 as an embryokine because IL6 improves ICM cell numbers in bovine blastocysts [51,52,53,54]. There was nearly a doubling in ICM cell numbers when supplementing 100 ng/mL recombinant bovine IL6 compared to non-treated (control) embryos [51,52,53]. Less impressive improvements in ICM cell numbers could be seen when providing 1 and 10 ng/mL IL6 [49]. Sang et al. did not detect a positive effect of IL6 on ICM numbers in bovine embryos [51]. They used an IL6 concentration of 50 ng/mL. This and other differences in embryo medium composition or other culture conditions may have contributed to the variable responses observed here. Further work is needed to determine how repeatable the IL6 effect is on ICM cells.

It remains unclear whether IL6 influences TE cell numbers [51,52,53,54], but it was clear that any increases in TE cell numbers that were detected were minor by comparison to the ICM cell responses. A more noteworthy function of the TE as it relates to IL6 function is the possibility that the TE may act as a barrier for IL6 provided to the culture medium. Tight junctions between TE cells usually restrict molecule movement through the blastocyst [53], but there is evidence in other epithelial borders throughout the body that IL6 can pass through tight junctions by using passive and active diffusion mechanisms [54,55]. Thus, it seems likely that medium-derived IL6 is directly responsible for the actions observed within the ICM. However, large IL6 concentrations (100 ng/mL) are needed to induce this effect, so perhaps only a fraction of the IL6 supplemented to the medium reaches the ICM. Further investigations are needed to define exactly how medium-supplemented IL6 influences the ICM.

Another action of IL6 is to preferentially promote the primitive endoderm (PE) cell lineage in IVP bovine blastocysts [56]. There is a stepwise reduction in PE cell numbers as the blastocyst ages in vitro from day 8 to 10 post-fertilization, but IL6 supplementation increases PE cell numbers within this time period. It is not clear if IL6 improves PE cell proliferation or limits PE cell apoptosis, but there is evidence suggesting that IL6 does not function as a lineage-specifying agent, where it would facilitate the conversion of naïve ICM cells into either epiblast (EPI) or PE cell lineages, but rather that it functions after those lineages have been established [56]. The effects of IL6 on the EPI cell lineage remain unclear. Our previous research detected increases in EPI cell numbers when counting NANOG^+^ nuclei, a marker for EPI cells [56]. However, another research group failed to detect changes in the number of NANOG^+^ cells after treatment with 50 ng/mL IL6 [51].

The promotion of ICM size and expansion of the PE lineage could have positive implications on pregnancy outcomes. The size and morphology of the ICM are primary morphological indicators of bovine and human IVP embryo quality [57,58]. There also is compelling evidence supporting the contention that bovine IVP blastocysts have fewer total ICM cells than their in vivo-produced counterparts [59,60,61,62], and this compromises the size of the embryonic disk, the structure that develops from the ICM that contains EPI, PE, and other embryonic and extraembryonic tissues [63]. In fact, several research groups have noted that embryonic disks cannot be detected via stereomicroscope in a subset of day 15–17 IVP bovine conceptuses [40,64,65,66,67]. Conceptuses with undetectable embryonic disks truly are inferior as they are unlikely to retain pregnancies when transferred back into cattle [64]. IL6-induced improvements in ICM size could potentially improve the size of the embryonic disk in IVP conceptuses.

Improvements in PE cell numbers in IL6-treated IVP embryos are noteworthy because the PE lineage produces the yolk sac. The yolk sac is indispensable in early pregnancy because of its role in processing nutrients from the uterine histotroph during early pregnancy [68,69]. Placentomes are not well established in bovine pregnancies until ~day 40 of pregnancy, and prior to this time, the conceptus must rely on histotroph for nourishment, which is digested by the yolk sac [70,71]. A link also exists between yolk sac development and pregnancy loss is a subset of IVP-generated pregnancies. Microscopic anomalies in yolk sac villous formation and vascular development have been observed in IVP pregnancies [72,73]. Moreover, pregnancy losses coincide with the time when yolk sac function is crucial for pregnancy maintenance. The yolk sac can be detected by day 12 of gestation and is maximal in size and vascularity between days 20–30 of pregnancy [73,74,75,76].

Our recent work suggests that IL6 treatment may correct the reductions in embryo-proper and fetus size normally observed in pregnancies generated from IVP embryos [52]. Reductions in crown-rump length and other parameters of the embryo/fetal size were noted in IVP pregnancies [52,77]. However, treatment with IL6 before ET generated embryos/fetuses that more closely resembled the size of embryos/fetuses in pregnancies derived from inseminated cows [52]. Also, a numerical improvement in pregnancy retention at day 70 of gestation was observed in IL6-treated embryos when compared with IVP control embryos (42% versus 26%, respectively).

The mechanism of IL6 action in bovine embryos appears to require Janus Kinase (JAK) and STAT3 signaling. The JAK-STAT3 signaling module is essential for ICM and PE cell maintenance in bovine embryos [50,78]. Exposure to a pharmacological inhibitor of JAK1/2 before the blastocyst is formed does not prevent blastocyst formation but greatly reduces total ICM cell numbers to only a fraction of their normal numbers [50,78]. Providing the inhibitor after blastocyst formation also reduces total ICM numbers, and nearly all of these cell losses are PE cells [56]. In many cases, no PE cells were observed in blastocysts after 48 h exposure to the inhibitor, whereas EPI cell numbers are not greatly affected by this treatment [56]. There is also evidence that IL6 acts through JAK-STAT3 in bovine embryos. Specifically, IL6 stimulates STAT3 phosphorylation and nuclear localization in all cells at the 9–16 cell stage, but this IL6-dependent activation occurs only within ICM cells at the blastocyst stage [50]. As EPI and PE specification occurs, IL6-induced STAT3 activation occurs only within the PE cell lineage [56]. Unfortunately, since STAT3 is crucial for ICM maintenance [50,78], one cannot directly test whether IL6 acts solely through STAT3 or if it utilizes alternative pathways to regulate ICM development.

Lastly, it is interesting to note that the actions of IL6 on the embryo are not restricted to cattle, but rather, IL6 also contains biological activities in other species. The embryonic action of IL6 has been explored in the pig, where increases in ICM cell numbers have been reported in porcine blastocysts following IL6 supplementation [79]. IL6 is produced throughout early embryogenesis in the pig, and it is preferentially produced within the ICM at the blastocyst stage [80,81]. Like in the cow, IL6 supplementation stimulates STAT3 phosphorylation in both mouse and pig blastocysts [79,82]. Moreover, antibody-based immunoneutralization of embryo-derived IL6 reduces STAT3 activation in mouse blastocysts [83]. There also are some indications that IL6 may facilitate embryonic stem cell (ESC) and induced pluripotent stem cell (iPSC) pluripotency [83,84]. There is a 50-fold increase in *IL6* mRNA during human fibroblast reprograming [85]. In the same report, IL6 supplementation stimulated several STAT3-induced targets, including *c-Myc*, one of the four factors required to induce pluripotency in human and mouse somatic cells [86]. Providing IL6 in place of *c-Myc* expression permitted cells to undergo pluripotency when the remaining three factors were overexpressed (*Oct4, Klf4, Sox2*).

## 5. Leukemia Inhibitory Factor

The positive effects of LIF on mouse embryo development and implantation are well described [87,88,89,90], but the effects of LIF on bovine embryos are less clearly defined. Several groups have reported the benefits of LIF supplementation on IVP bovine blastocyst formation rates [27,91,92], whereas other groups saw no benefits of LIF supplementation [91,92,93,94]. No studies have detected a positive effect of LIF supplementation on ICM cell numbers and PE development in bovine blastocysts [95,96,97]. It also is unclear whether LIF functions as a cryo-survival agent in bovine embryos [91,92,93,94,95,98]. At least some and perhaps a substantial portion of the disparity in these responses may have resulted from the wide range in LIF concentrations used (500–6000 IU [2–100 ng/mL]) and from the use of human and mouse recombinant proteins. Another contributing factor to these variable responses is the low-level expression of the LIF receptor (*LIFR*) during early embryonic development. One report found that *LIFR* transcripts could be detected in IVP bovine embryos whereas they were absent in in vivo produced embryos [97], suggesting that LIF actions may not exist unless embryos are being produced in vitro. Our laboratory detected *LIFR* mRNA in IVP bovine blastocysts, but the abundance of this transcript was 50-fold less than that of *IL6R* [50].

There is evidence that supports including LIF as an embryokine when considering its actions after blastocyst formation. The most convincing evidence of LIF acting as an embryokine has been reported in the mouse where LIF supplementation promotes PE cell development [98] and improves post-transfer implantation success [21]. Moreover, *Lifr* loss-of-function mutation produces a partial embryonic lethal phenotype in mice with fewer *Lifr*^-/-^ embryos in litters from the blastocyst stage onward [99], suggesting that the loss of *Lifr* reduces embryo survivability. It is uncertain how these observations made in the mouse may relate to the bovine embryo. No work exists that has explored the actions of LIF on or after day 9 of IVP bovine embryo development.

Further investigation is needed to explore whether LIF serves as a pluripotency factor in bovine stem cells. There is ample evidence for LIF as a pluripotency factor in ESCs and iPSCs, and specifically that LIF is the primary ligand used to support naïve-state pluripotency in the pig, human, monkey, and mouse by stimulating the IL6ST-JAK-STAT3 signaling cascade [100,101]. It is not clear if LIF is needed to establish naïve-state pluripotency in bovine stem cells. To date, only bovine ESCs that resemble primed-state stem cells have been generated [102,103].

## 6. Other Cytokines That May Serve as Embryokines

The embryokine actions of CSF2 have received most of the attention within the β-common cytokine family, but some attention has been given to exploring whether IL3 also serves as an embryokine. It is expressed within the ovine endometrial epithelium during diestrus [104], but the sole study exploring IL3 effects on IVP bovine embryos failed to detect a positive effect of IL3 on cleavage or blastocyst rates [105]. However, there is evidence that IL3 increases *IFNT* production in ovine conceptuses [104]. No studies have explored whether IL5 acts on bovine or ovine embryos and conceptuses.

The only other cytokines we are aware of that have been explored as embryokines in bovine IVP embryos are interleukin-1α (IL1A), interleukin-1ß (IL1B), IL8, and stem cell factor (SCF, Kit ligand). Each of these ligands is expressed either by the endometrium or the embryo itself [48,106,107], and receptors, co-receptors, and receptor inhibitors for these cytokines have been detected in bovine embryos [46,50,51,108] (see Table 1). No definitive role for SCF as an embryokine has been found thus far. Positive effects of IL1B supplementation on bovine IVP development have been noted, although these positive effects were only detected when embryos were cultured in large groups, suggesting that IL1B may be acting indirectly by stimulating the production of an embryo-derived embryokine [109]. Supplementation with IL8 tended to improve blastocyst hatching but decreased ICM cell numbers [51].

Transcriptome profiling studies may offer new insights into additional cytokines that may be involved with early embryogenesis in cattle. Table 1 provides information about cytokine transcripts detected in the bovine endometrium at days 5 and 7 post-estrus [48] and in IVP bovine blastocysts harvested at day 8 post-fertilization [50]. A listing of cytokine receptor transcripts detected in the day 8 bovine blastocysts is also included [50].

Several interesting observations were detected when examining these data. First, several chemokines were identified in the bovine endometrium and blastocyst (abbreviated with the CXCL and CCL designations), but only one chemokine receptor was detected in bovine blastocysts (*CCR10*). It remains unknown if the chemokines that react with this receptor (*CCL27*, *28*) [110] are produced by the bovine endometrium as these chemokines were not included in the endometrium study referenced in Table 1 [48]. Thus, it appears that many of the endometrial and blastocyst chemokines are not acting on bovine embryos, or at least not at the blastocyst stage, but it will be interesting to learn whether CCR10 receptor activation or inhibition plays any role in early embryogenesis in cattle.

Our global transcript profiling work in IVP bovine blastocysts detected several cytokines and cytokine receptors (Table 1) [50]. Arguably the most notable finding was observing several α-subunit receptors for members of the IL6 cytokine family (*IL6R*, *IL6ST*, *IL11RA*, *CNTFR*, and *LIFR*) [50]. The presence of *IL11R* in bovine blastocysts is particularly interesting because IL11 is expressed in bovine endometria, and its expression is greatest at day 7 post-estrus, coincident with blastocyst formation and initial ICM development in in vivo-generated bovine embryos [111]. The presence of *CNTFR* in bovine blastocysts may indicate that CNTF is biologically active in the embryo during early pregnancy, although there is no evidence of CNTF expression by either the endometrium or embryo during early pregnancy.

## 7. Concluding Remarks

**Figure 2:**
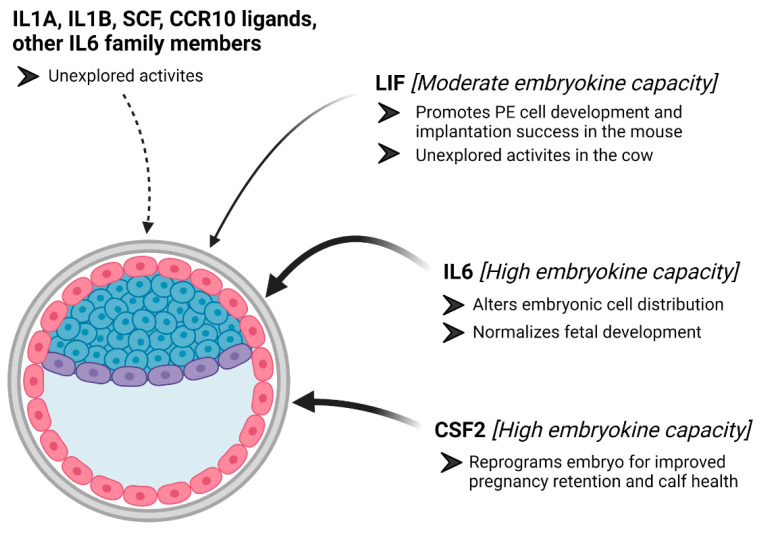
Model of our current knowledge about embryokine actions for cytokines. Colony-stimulating factor 2 (CSF2) and interleukin 6 (IL6) contain embryokine activity in bovine embryos (an illustration of a bovine embryo is provided). Leukemia inhibitory factor (LIF) may contain embryokine activity, but this activity is suggested namely because of embryokine actions for LIF in the mouse embryo. Several other potential cytokines may serve as embryokines, but work is needed to describe these potential activities. The figure was created with BioRender.com.

In the cow, there is convincing evidence that CSF2 serves as an embryokine, and recent evidence from our group supports the notion that IL6 should also be categorized as an embryokine (Figure 2). Both of these molecules are distinct from many of the other embryokines that have been studied because neither molecule consistently improves blastocyst formation rates in IVP bovine embryos. Rather, CSF2 appears to reprogram the IVP embryo, so it has greater survivability after ET and so that fetuses and calves more closely resemble those generated by AI. The actions of IL6 appear to be associated with modifying the cellular composition of the blastocyst in ways that we predict could improve pregnancy retention after ET and adjust fetal development, so it more closely resembles fetal development in AI-derived pregnancies. More work is needed to describe how LIF, IL1A, IL1B, SCF, and CCCR10 ligands may function as embryokines in cattle. Available evidence suggests that these molecules serve some purposes during embryogenesis, and it will be interesting to explore whether these activities may improve bovine embryo competency.

Two definitive conclusions that can be made at this point: (1) it is clear that some cytokines are serving as embryokines in cattle, and (2) the cytokines studied thus far appear to function primarily by improving IVP bovine embryo quality and competency rather than by increasing the number of transferrable IVP embryos. Further investigations are necessary to provide mechanistic knowledge of how these and other cytokines function as embryokines. Another gap in our knowledge is exploring other cytokines as embryokines. Cytokines that should be considered in future research include IL1A, IL1B, SCF, CCR10 ligands, and other IL6 family members.

## Figures and Tables

**Table 1:** Cytokines expressed by the bovine endometrium and cytokines and cytokine receptors expressed by IVP bovine blastocysts.

Cytokine Transcripts Endometrium ^1^	Cytokine Transcripts Blastocyst ^2^	Cytokine Receptor Transcripts Blastocyst ^2^
*CXCL3*	*CCL17*	*IL6R*
*IL8*	*IFNT*	*IL6ST*
*CXCL12*	*IL18*	*IL2RB*
*CSF2*	*IL1RN*	*IL10RB*
*IL1A*	*CXCL5*	*IL13RA*
*CXCL10*	*CCL24*	*IFNGR*
*CX3CL1*	*IL6*	*IL36RN*
*CXCL16*	*CXCL16*	*IFNAR*
*CCL14*	*IL27*	*IL11RA*
*IL18*	*CTF1*	*IL17RA*
*IL33*	*CLCF1*	*IL20RB*
*IL6*		*IL20RA*
*IL16*		*IL1R1*
*IL12A*		*IL17RC*
*IL12B*		*CSF2RA*
*IL34*		*CCR10*
*IL1B*		*CNTFR*
*CCL21*		*LIFR*
*IL15*		*IL1RAP*

Data taken from ^1^ [48] and ^2^ [50]. Transcripts are ranked from greatest to least expression within each column. The endometrial expression represents transcript abundance of 93 genes at days 5 and 7 post-estrus using the NanoString nCounter Analysis System (NanoString Technologies, Seattle, WA, USA). Values were normalized to RNA spike-in controls and 6 housekeeping genes [48]. The blastocyst data represent RNA-sequencing analysis (Illumina Platform) of bovine IVP blastocysts harvested at day 8 post-fertilization. Data are represented as reads per kilobase of transcript per million transcripts (RPKM) [50]. Only RPKM values that were greater than 0.5 are presented.

## Data Availability

Not applicable.
